# C5a Regulates IL-12^+^DC Migration to Induce Pathogenic Th1 and Th17 Cells in Sepsis

**DOI:** 10.1371/journal.pone.0069779

**Published:** 2013-07-23

**Authors:** Ning Ma, Chen Xing, He Xiao, Yi Wang, Ke Wang, Chunmei Hou, Gencheng Han, Guojiang Chen, Bernadette Marrero, Yujuan Wang, Beifen Shen, Yan Li, Renxi Wang

**Affiliations:** 1 Laboratory of Immunology, Institute of Basic Medical Sciences, Beijing, China; 2 Department of Rheumatology, First hospital of Jilin University, Changchun, China; 3 Molecular Immunology Section, Laboratory of Immunology, National Eye Institute, National Institutes of Health, Bethesda, Maryland, United States of America; 4 Immunopathology Section, Laboratory of Immunology, National Eye Institute, National Institutes of Health, Bethesda, Maryland, United States of America; University of Cincinnati, United States of America

## Abstract

**Objective:**

It is well known that complement system C5a is excessively activated during the onset of sepsis. However, it is unclear whether C5a can regulate dentritic cells (DCs) to stimulate adaptive immune cells such as Th1 and Th17 in sepsis.

**Methods:**

Sepsis was induced by cecal ligation and puncture (CLP). CLP-induced sepsis was treated with anti-C5a or IL-12. IL-12^+^DC, IFNγ^+^Th1, and IL-17^+^Th17 cells were analyzed by flow cytometry. IL-12 was measured by ELISA.

**Results:**

Our studies here showed that C5a induced IL-12^+^DC cell migration from the peritoneal cavity to peripheral blood and lymph nodes. Furthermore, IL-12^+^DC cells induced the expansion of pathogenic IFNγ^+^Th1 and IL-17^+^Th17 cells in peripheral blood and lymph nodes. Moreover, IL-12, secreted by DC cells in the peritoneal cavity, is an important factor that prevents the development of sepsis.

**Conclusion:**

Our data suggests that C5a regulates IL-12^+^DC cell migration to induce pathogenic Th1 and Th17 cells in sepsis.

## Introduction

Although there are presently better broad spectrum antibiotics and new therapies available, sepsis is still a severe disease that is associated with high mortality [1], [2]. Many cytokines are largely produced during sepsis and it is believed that the simultaneous release of all kinds of cytokines is strongly related with pathogenesis of sepsis.

During the onset of sepsis, it is well known that the complement system is excessively activated through three pathways known as the classical pathway, alternative pathway and lectin pathways [3]. Among the complement activated products, C5a act as a potent chemoattractant. C5a has a number of functions including modulation of cytokines expression [4] causing oxidative burst and granule enzymes [5]–[7] and improving the expression of adhesion molecules of neutrophils [8]. C5a is harmful to mice after CLP under unregulated conditions [9] which results in inhibiting H2O2 production from neutrophils [10]; causing reduced neutrophil apoptosis and enhanced thymocyte apoptosis [11]–[13] excessively enhancing proinflammatory cytokine production [14]–[17]. All these studies suggest that C5a plays a critical role in the innate immune response.

A recent publication shows that C5a can also regulate adaptive immune responders in particularly regulatory T cells [18]. Dendritic cells (DCs) are the principle antigen presenting cells (APC) and central components of the host’s innate immune system. DCs mature once stimulated by microbes and produce large amounts of Th1 cytokine IFNγ [19]–[22]. However, it is unclear whether C5a can directly regulate DC cells to stimulate adaptive immune cells such as Th1 and Th17 in sepsis. Our current study showed that C5a induced IL-12^+^DC cell migration from the peritoneal cavity to peripheral blood and lymph nodes. IL-12^+^DC cells induced pathogenic IFNγ^+^Th1 and IL-17^+^Th17 cells in peripheral blood and lymph nodes, whereas IL-12, secreted by DC cells in the peritoneal cavity, protected against sepsis.

## Materials and Methods

### Ethics Committee Approval

Care, usilization and treatment of mice in this study were in strict agreement with international guidelines for the care and use of laboratory animals and also approved by Animal Ethics Committee of Beijing Institute of Basic Medical Sciences.

### Mice

Seven to eight-week-old male C57BL/6 mice and conditional DC-depleted B6.FVB-Tg (Itgax-DTR/EGFP)57Lan/J mice were obtained from the Jackson laboratory (Bar Harbor, ME, USA) and bred in our facilities under specific pathogen-free conditions.

### Production of Anti-C5a Antibody

The C-terminal end of mouse C5a (sequence: CTIANKIRKESPHKPVQLGR) corresponding to amino acids 58–77 was chosen for peptide synthesis. The peptide was coupled to keyhole limpet and used for the immunization of rabbits and production of anti-C5a. The polyclonal antibody was purified by protein A chromatograph, and its reactivity with recombinant mouse C5a (Hycult biotechnology b.v, uden, The Netherlands) was confirmed by ELISA.

### Lysozyme Release Assay

100 nM mouse C5a (Sigma-Aldrich) and 100 nM preimmune IgG (JingMei Biotechonogy, Beijing, China) or anti-C5a were incubated for 2 hours at room temperature. Peripheral blood cells (PBMC) were collected from 7-week-old mice and diluted in 2 times the volume of whole blood. PBMC were incubated for 5 min with cytochalasin B (Sigma-Aldrich) at 37 and then stimulated for 15 min with 100 nM mouse C5a or preincubated mixture (100 nM mouse C5a and 100 nM preimmune IgG, or 100 nM mouse C5a and 100 nM anti-C5a) and incubated at 37°C. 50 µl cell-free supernatant was collected and mixed with 50 µl 2 mM 4-nitrophenyl N-acetyl-b-glucosaminide (Sigma-Aldrich) for 1 hour at 37°C. The reaction was stopped by adding 150 µl 0.1 M pH 9.5 Na_2_CO_3_/NaHCO_3_ buffer. Fluorescence was read by absorption maxima at 485 and emission maxima at 530.

### Induction of Sepsis by CLP

Specific pathogen-free 7–8 week old male B6.FVB-Tg (Itgax-DTR/EGFP)57Lan-J mice were treated with DTT 18 to 20 h prior to performing the CLP procedure and C57BL/6 mice were used for studies as indicated. Sepsis was induced by Cecal Ligation and Puncture (CLP), the severity of sepsis was highly dependent on the extent of cecal ligation. Briefly, for the induction of mild-grade sepsis, which resulted in survival rates of ∼50%, the cecum was ligated at half the distance between distal pole and the base of the cecum. While high-grade sepsis (25% survival rates) involves ligation of 75% ligation of the cecum. After the bowel was sectioned, ligated and repositioned, the abdomen was closed in layers by using a 4.0 surgical suture and metallic clips. Sham-operated mice were handled in the same manner, except that the cecum was not ligated and punctured. After the CLP procedure, each mouse was intravenously injected with 40 µg of anti-C5a IgG in 100 µL Dulbecco’s phosphate buffered saline solution (DPBS). Control animals were injected with the same amount of normal rabbit IgG (JingMei Biotechonogy, Beijing, China).

### Cytometric Analysis and Intracellular Cytokine Staining

Cells that were collected and isolated from the mice (1 × 10^6^ cells/sample) were washed with fluorescence-activated cell sorting (FACS) staining buffer (phosphate-buffered saline, 2% fetal bovine serum or 1% bovine serum albumin, 0.1% sodium azide). All samples were incubated with the 2.4G2 anti-Fc receptors (BD Pharmingen), prior to incubating with other fluorescently conjugated Abs diluted in FACS buffer supplemented with 2% anti-Fc receptor Ab. For intracellular cytokine staining, 50 ng/mL PMA and 1 mg/mL Ionomycin (all from Sigma-Aldrich) were added and 3 hours later, inhibitors of protein secretion 1 mg/mL brefeldin A and 2 mM monensin were added during the incubation period. Cells were collected and fixed for 20 min with 1 mL fixation buffer (Fix and Perm cell permeabilization kit, eBioscience). After washing, the fixed cells were stained. The following antibodies were purchased from eBioscience: anti-mouse CD4, CD11c, IL-12, IFNγ, and IL-17 antibody. Data collection was performed on a FACS Calibur flow cytometer and the data were analyzed using CellQuest software.

### Cytokine Analysis by ELISA

The concentration of IL-12 was measured by ELISA kits (eBioscience). Briefly, 96-well microtiter plates were coated with 96-well microtiter plates were coated with purified rat anti-mouse IL-12 p70 (4 µg/ml) overnight at 4°C. Then the plates were blocked with 10% FCS for 2–3 h at 37°C. After washing, supernatants were added to the plate in triplicate for 1 h at 37°C. After washing, biotin rat anti-mouse IL-12 p70 (4 µg/ml) Abs were added to the plates and incubated for another hour at 37°C. Thereafter, unbound Abs were washed off, followed by the addition of avidin-HRP (1/1000 dilution) (all Abs were obtained from eBiosciece). Plates were incubated for 1 h at 37°C. Finally, the color was developed by incubation with an o-phenylenediamine substrate. The OD was read at 492 nm with an ELISA plate reader (Bio-Rad). Standard curves were established to quantitate the amount of the respective cytokines.

### Depletion of DCs

To deplete CD11c^+^ DC, conditional DC-depleted mice were treated with an intraperitoneal (i.p.) injection of 8 ng/g body weight of diphtheria toxin (DT) (Sigma-Aldrich, St. Louis, MO, USA). As controls, conditional DC-depleted mice were injected i.p.with saline vehicle alone. All the mice underwent the CLP procedure 18 to 20 h after i.p. injections.

### Statistical Analysis

Results of flow cytometry and cytokine ELISA were analyzed by the Students t-test. The Kaplan–Meier method was utilized to analyze actuarial overall survival rates. All differences reported in the results were significant (*P<0.05, **P<0.01, ***P<0.001, ****P<0.0001).

## Results

### Anti-C5a Reduced IL-12^+^DC Cells in PBMC and LN of Septic Mice

**Figure 1:**
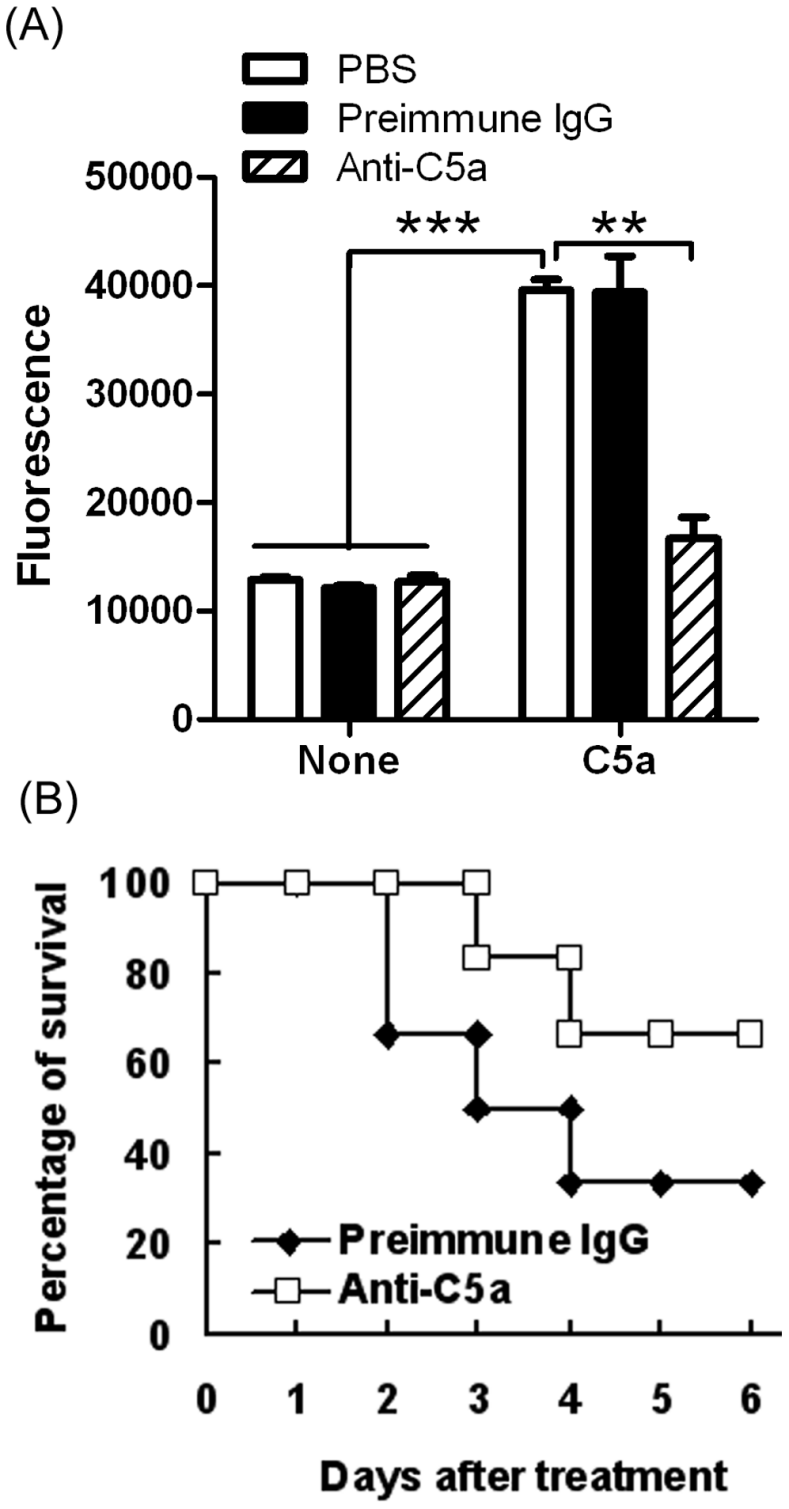
Anti-C5a treatment ameliorated sepsis. (**A**) **Anti-C5a antibody blocked the C5a effect.** 100 nM mouse C5a and 100 nM preimmune IgG or anti-C5a were incubated for 2 hours at room temperature. Peripheral blood cells (PBMC) were collected from 7-week-old mice and diluted 2 times the volume of whole blood. PBMC were stimulated for 15 min at 37°C with 100 nM mouse C5a or preincubated mixture (100 nM mouse C5a and 100 nM preimmune IgG, or 100 nM mouse C5a and 100 nM anti-C5a). Fluorescence was determined by an enzyme reaction processed from cultured supernatants described in Materials and Methods. The results were analyzed based on three independent experiments (**P<0.01, ***P<0.001). (**B**) **Anti-C5a antibody ameliorated sepsis.** Peritonitis was induced in C57BL/6 mice by cecal ligation and puncture (CLP). Immediately after CLP operations, each mouse was intravenously injected with 40 µg of anti-C5a IgG in 100 µL Dulbecco’s phosphate buffered saline solution (DPBS). Control animals received similar a amount of normal rabbit IgG. Data are presented as the percentage of survival (n = 9/group). Overall survival rates were analyzed by the Kaplan–Meier method. Compared to isotype IgG antibody-treated littermates, the anti-C5a antibody-treated mice showed a higher survival rate (p<0.05).

Sepsis models were made by cecal ligation and puncture (CLP). Here both mild grade sepsis (with a survival rate of about 50%) and high-grade sepsis (with a survival rate of about 25%) models, as described in the materials and methods, were used. Unless otherwise mentioned, CLP mice referred to mice with high-grade sepsis. To prove that there is an association between the critical inflammatory cytokines C5a and DCs in CLP-induced sepsis, a polyclonal anti-C5a antibody was developed. C5a-induced lysozyme release assay showed that anti-C5a antibody effectively blocked the effects of C5a (Fig. 1A). CLP-induced septic mice were intravenously injected with anti-C5a antibody or a similar dose of nonspecific IgG antibody. The anti-C5a antibody-treated mice showed significantly higher survival rates compared with isotype IgG antibody-treated littermates (p<0.05, Fig. 1B). These results indicate that anti-C5a antibodies can effectively treat sepsis.

**Figure 2:**
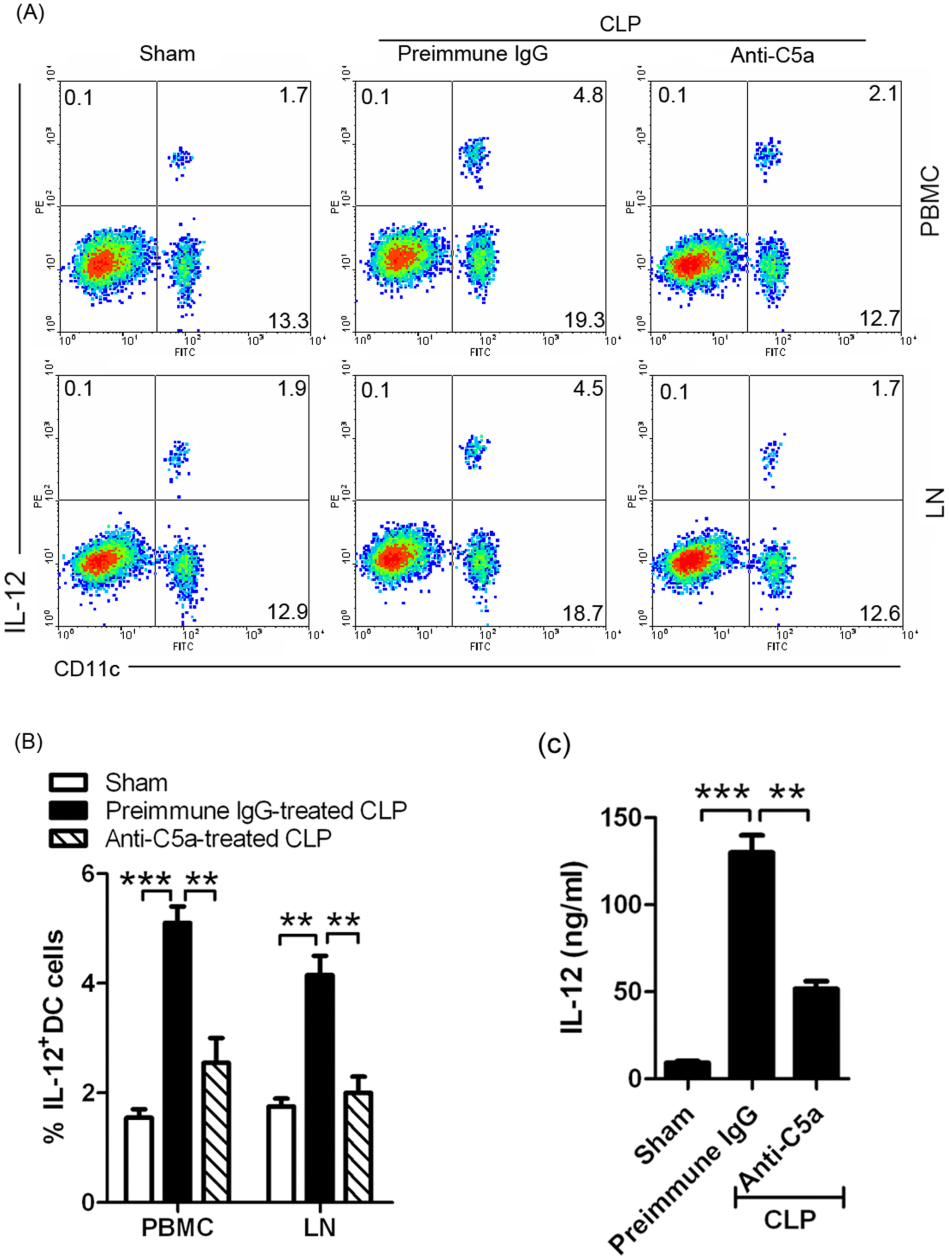
Anti-C5a reduced IL-12^+^DCs in PBMC and LN. (**A and B**) **IL-12^+^DCs cells decreased in PBMC and LN from anti-C5a-treated sepsis.** (A) Mononuclear cells were collected by Ficoll solution from PBMC and LN in sham, preimmune IgG-treated CLP, or anti-C5a-treated CLP mice (described as Fig. 1) on day 3 after sepsis induction. Cells stained with anti-mouse CD11c and IL-12 with numbers in quadrants indicating the percentage of IL12^+^DC cells. (B) The statistical analysis was done based on four independent experiments (**P<0.01, ***P<0.001). (**C**) **IL-12 level decreased in PBMC from anti-C5a-treated sepsis.** The serum was collected from sham, preimmune IgG-treated CLP, or anti-C5a-treated CLP mice (described as Fig. 1) on day 3 after sepsis induction. IL-12 level in the serum was determined by ELISA. The statistical analysis was done based on three independent experiments (**P<0.01, ***P<0.001).

Because IL-12^+^DC cells were the important antigen presenting cells (APCs) which promote inflammatory responses, we first tested for the presence of IL-12^+^DC cells using FACS analysis. On day 3 after sepsis induction, mononuclear cells were collected by Ficoll solution from PBMC and LN in different treatment groups. The results demonstrated that sepsis induced IL-12^+^DC cells in PBMC and LN and anti-C5a-treatment could efficiently reduce IL-12^+^DC cells (Fig. 2A and B). Furthermore, we found that IL-12 levels were decreased in PBMC from anti-C5a-treated mice (Fig. 2C). These results suggest that anti-C5a antibody reduced IL-12^+^DC cells in PBMC and LN of septic mice.

### IL-12^+^DC Cells Increased in the Peritoneal Cavity of Anti-C5a-treated Septic Mice

**Figure 3:**
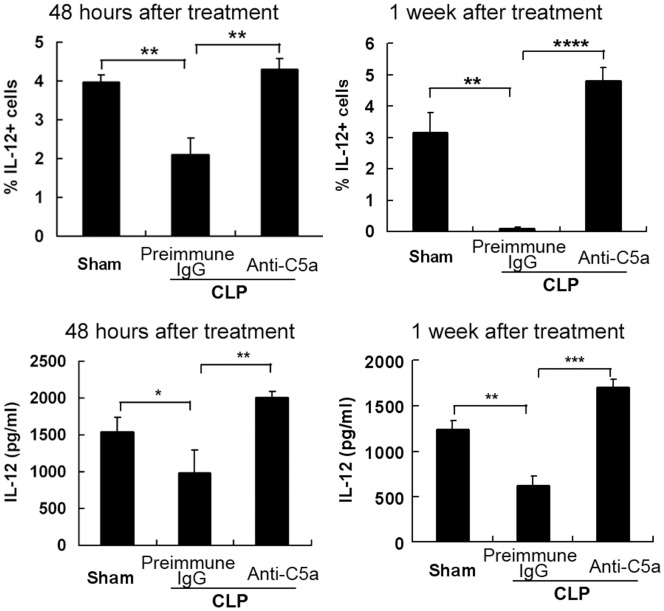
IL-12^+^DCs increased in peritoneal cavity of anti-C5a-treated CLP mice. Peritonitis was induced in C57BL/6 mice by CLP. We chose 2 time points: 48 hours and 1 week after induction of sepsis. Normal saline was injected to the peritoneal cavity of mice and washed several times. Cell-containing fluid was collected. Peritoneal cells were stained with PE-anti-IL-12 mAb to detect the percentage of IL-12 positive cells on the gate of monocytes population. The percentage of IL-12 positive cells was analyzed (upper panel). The peritoneal fluid was collected and IL-12 level was determined by ELISA (lower panel). The statistical analysis was done based on three independent experiments (*P<0.05, **P<0.01, ***P<0.001, ****P<0.0001).

We chose 2 time points to analyze the samples: 48 hours and 1 week after induction of sepsis. Cell-containing fluid was collected from peritoneal cavity. Peritoneal cells were stained with PE-anti-IL-12 antibody to detect the percentage of IL-12^+^DC cells gated on the monocyte population. The percentage of IL-12 positive cells was also analyzed (Fig. 3, upper panel). Compared with preimmune-treated septic mice, mice received anti-C5a antibody have a high percentage of IL-12-expressing cells. Furthermore, the level of IL-12 obtained from the peritoneal fluid was determined by ELISA (Fig. 3, lower panel). The result demonstrated that the level of IL-12 was higher in C5a treatment than that in preimmune IgG-treated sepsis. These results in Figure 2 and Figure 3 together suggest that IL-12^+^DC cells were detained in the peritoneal cavity from anti-C5a treated mice.

### Sepsis Reduced IL-12^+^DC Cells in Peritoneal Cavity

**Figure 4:**
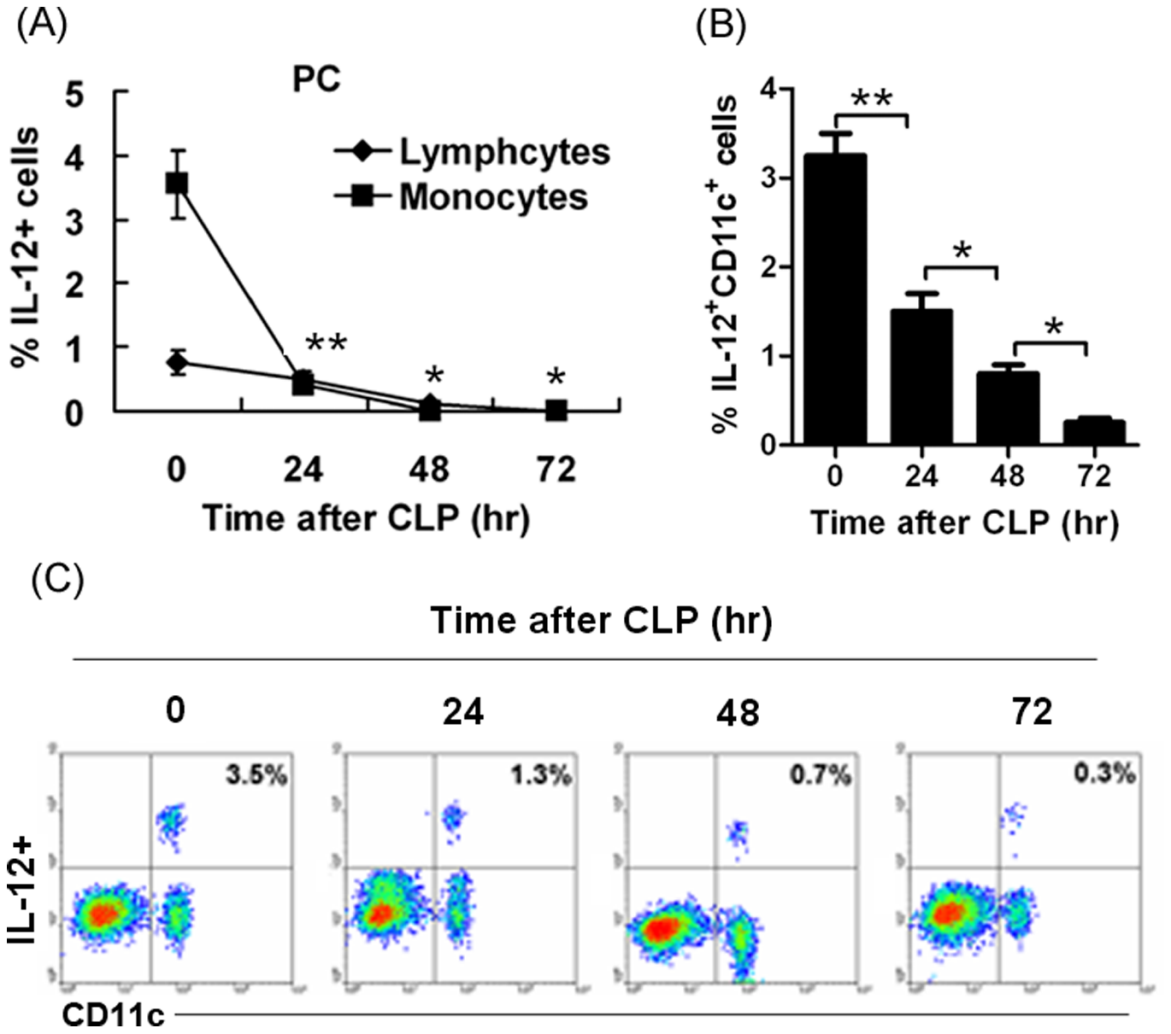
Sepsis reduced IL-12-expressing cells in peritoneal cavity. (**A**) **Sepsis dramatically and time-dependently reduced IL-12^+^ cells in peritoneal cavity.** Peritonitis was induced in C57BL/6 mice by CLP. We chose 4 time points: 0, 24, 48, 72 hours. Normal saline was injected into the peritoneal cavity of mice and washed several times. Cell-containing fluid was collected. The peritoneal cells were stained with PE-anti-IL-12 mAb to detect percentage of IL-12 positive cells on the gate of lymphocyte and monocyte populations (*P<0.05, **P<0.01). (B-C) Sepsis dramatically and time-dependently reduced IL-12^+^CD11c^+^ cells in the peritoneal cavity. The peritoneal cells were also stained with PE-anti-IL-12 mAb and FITC-anti-CD11c mAb to detect whether DC cells expressed IL-12. The percentage of IL-12 positive cells was analyzed and compared between the groups. (B) The statistical analysis was done based on four independent experiments (*P<0.05, **P<0.01). (C) showed the percentage of IL-12^+^CD11c^+^ cells.

C57BL/6 mice were induced to peritonitis by CLP. We analyzed the cells from the peritoneal cavity at 0, 24, 48, 72 hours after CLP-induced sepsis. The percentage of IL-12 positive cells was determined by flow cytometry. Lymphocyte and monocyte population were gated, respectively. IL-12-expressing cells in monocytes population were higher than that in lymphocytes population in non-septic mice. When sepsis was induced, IL-12-expressing cells were dramatically and time-dependently reduced (Fig. 4A). The data suggest that IL-12 production was inhibited and may have protective properties against sepsis. Furthermore, we analyzed whether DCs expressed IL-12. By staining with the DC marker CD11c, we demonstrated that IL-12 was mainly expressed by DC cells (Fig. 4C) but in septic mice IL-12^+^CD11c^+^ cells from peritoneal cavity showed a dramatic reduction and differences were detected in a time-dependent manner (Fig. 4B and C).

### IL-12^+^DC Cells in the Peritoneal Cavity Play a Protective Role in Sepsis

**Figure 5:**
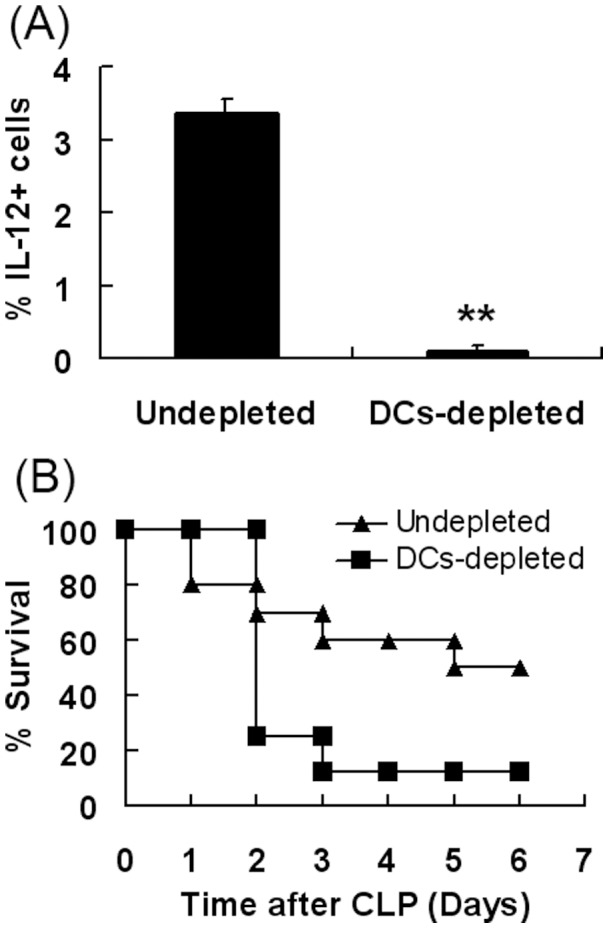
DC-depletion exacerbated the sepsis disease. (**A**) **IL-12-expressing cells were mainly DCs.** Conditional DCs-depleted mice B6.FVB-Tg (Itgax-DTR/EGFP)57Lan/J mice were developed by administering 8ng/g of diphtheria toxin (DT) by i.p. injection. Normal saline was injected into the peritoneal cavity of mice and washed several times. Cell-containing fluid was collected. Peritoneal cells were stained with PE-anti-IL-12 mAb to detect the percentage of IL-12 positive cells on the gate of monocyte population. The percentage of IL-12 positive cells was analyzed. The data represents at least four independent experiments (**P<0.01). (**B**) **DCs-depletion exacerbated the sepsis induced by CLP.** Conditional DCs-depleted mice were injected i.p. with 8ng/g of DT. On day 2, peritonitis were induced in DC-depleted mice by CLP. Data is presented as percentage survival (n = 9/group). Overall survival rates were analyzed by the Kaplan–Meier method. Compared with the wild type group, DCs-depleted mice have a higher mortality rate (P<0.05).

To deplete DCs, conditional DCs-depleted mice were injected i.p. with 8 ng/g of diphtheria toxin (DT). Cells-containing fluid was collected from peritoneal cavity. To detect the percentage of IL-12 positive cells on the gate of monocyte population, peritoneal cells were stained with PE-anti-IL-12 antibody. The percentage of IL-12 positive cells was analyzed by FACS analysis (Fig. 5A). Compared to the control, the IL-12-expressing cells decreased in DCs-depleted mice. The data indicated that DCs were the main source of IL-12 in peritoneal cavity.

Next, we examined whether IL-12-secreting DC cells have a protective role in sepsis. On day 2 after DCs depletion, peritonitis was induced in mice by CLP. Subsequently, mice were monitored every day. The data shows that DCs-depleted mice resulted in higher mortality rates compared to the wild type mice (Fig. 5B). In addition, the data demonstrated that IL-12-secreting DCs were essential for the survival of CLP mice.

### IL-12^+^DC Cells in PBMC Induced Pathogenic Th1 and Th17 Cells in Sepsis

**Figure 6:**
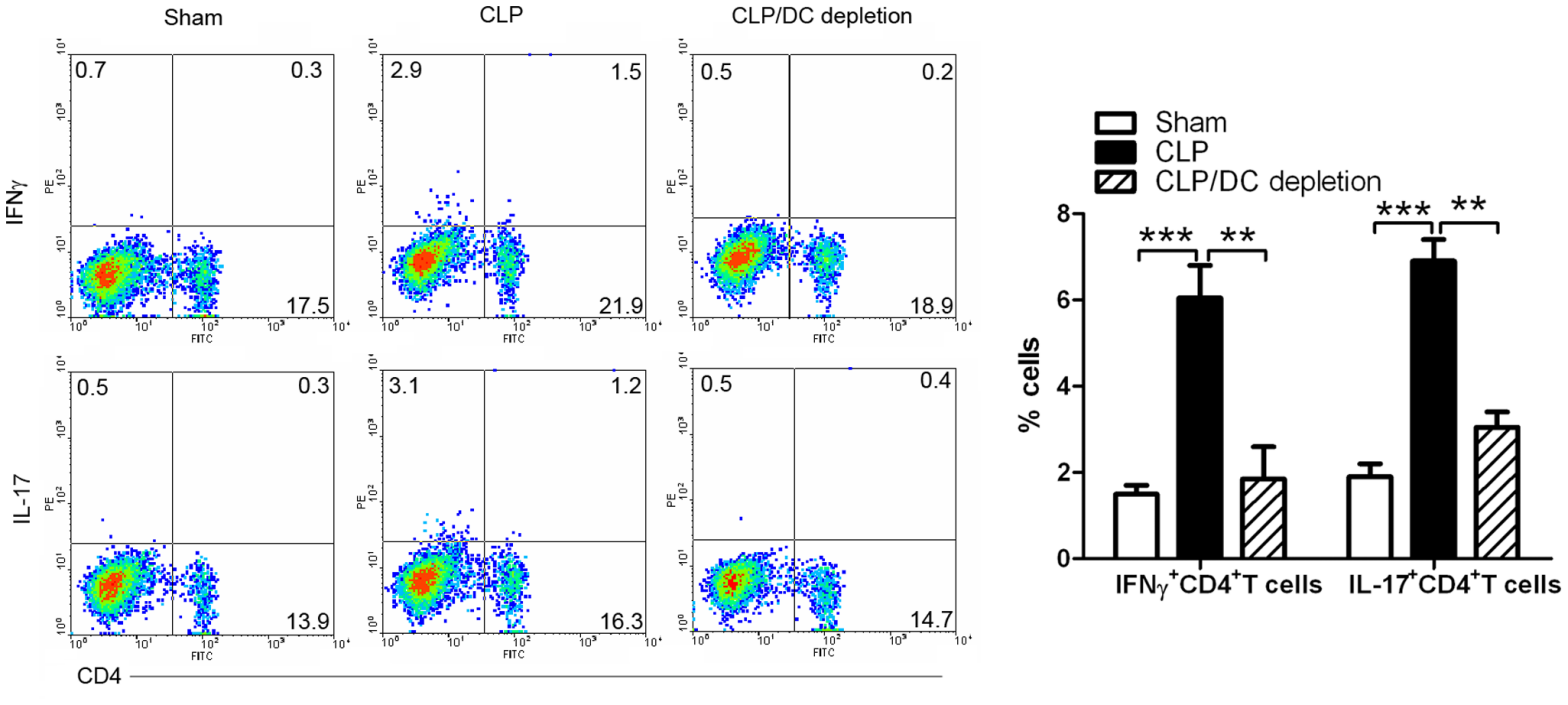
CD11c^+^DC cells induced Th1 and Th17 cells in sepsis. Lymphocytes were collected from PBMC in sham, CLP, CLP/DCs-depletion mice on day 3 after sepsis induction. Cells were stained with anti-mouse CD4, IFNγ, and IL-17 with numbers in quadrants indicating percentage of IFNγ^+^Th1 and IL-17^+^Th17 cells. Right panel showed the statistical analysis based on four independent experiments ((**P<0.01, ***P<0.001).

Th1 and Th17 cells are two important inflammatory adaptive immune cells. Herein, we detected whether IL-12^+^DC cells had an important role in inducing Th1 and Th17-cell production. To deplete DCs, conditional DCs-depleted mice were injected i.p.with 8 ng/g of DT. On day 3 after sepsis induction, lymphocytes were collected from PBMC. Cells were stained with anti-mouse CD4, IFNγ and IL-17 antibodies to analyze the percentage of IFNγ^+^Th1 and IL-17^+^Th17 cells. Compared to DCs-undepleted mice, DC-depleted mice significantly reduced IFNγ^+^Th1 and IL-17^+^Th17 cells. CLP-induced sepsis significantly up-regulated IFNγ^+^Th1 and IL-17^+^Th17 cells in DC-undepleted mice, compared with the sham control. The data obtained from LN was similar to that of in PBMC (data not shown). This data suggests that IL-12^+^DC cells in PBMC and LN induced pathogenic Th1 and Th17 cells in sepsis (Fig. 6).

### IL-12 Injection into the Peritoneal Cavity Prevented Disease in CLP Mice

**Figure 7:**
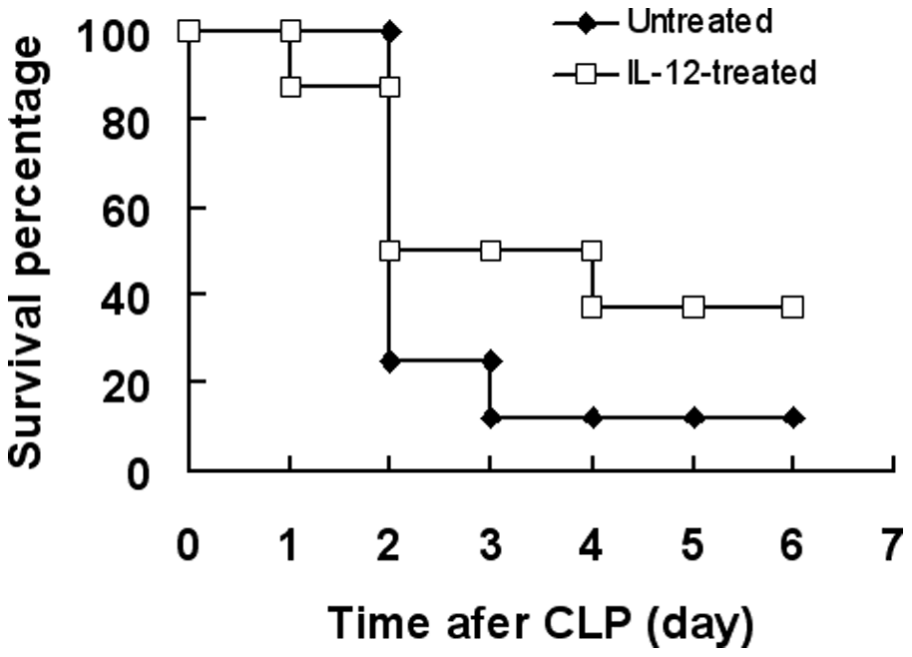
Injection of IL-12 in peritoneal cavity ameliorated sepsis. Conditional DC-depleted mice were injected i.p. with 8 ng/g of DT. On day 2, peritonitis was induced in DC-depleted mice by CLP. DC-depleted CLP mice were divided into two groups: one was given recombinant mouse IL-12 i.p.with injection, while the other group received normal saline as control. Data is presented as the percentage of survival (n = 9/group). Overall survival rates were analyzed by the Kaplan–Meier method. Compared with PBS-treated group, IL-12-treated group had a higher survival rate (P<0.05).

Peritonitis was induced in DC-depleted mice by CLP. We gave additional IL-12 to the peritoneal cavity of mice every day after CLP induction. IL-12-treated group had a higher survival rate than that in PBS-treated group (Fig. 7). The results suggest that injection of IL-12 in peritoneal cavity plays a protective role in sepsis.

## Discussion

Sepsis is a potentially deadly disease characterized by a systemic body inflammatory response. It is triggered through an infection [23], [24]. Caecal ligation and puncture (CLP) model of sepsis is believed to closely simulate clinical sepsis in humans through the polymicrobe-driven inflammatory process [25]. Severe sepsis represents the systemic inflammatory response, infection and the presence of organ dysfunction. Our data here show that sepsis induced an amount of inflammatory Th1 and Th17 adaptive cells (Fig. 6).

This inflammatory response is caused by the immune system to microbes in the blood, urine,lungs, skin, or other tissues. Dendritic cells (DCs) are the principal APCs that make up the central components of the host’s innate immune system. DCs undergo maturation when stimulated by microbial products and produce large amounts of Th1 cells [26], [27]. Our results showed that IL-12^+^DC cells were a key inducer of IFNγ^+^Th1 and IL-17^+^Th17 cells (Fig. 6). We found that sepsis caused IL-12^+^DC cells reduction in the peritoneal cavity and their up-regulation in PBMC and LN (Fig. 2–4). These results suggest that sepsis induced IL-12^+^DC cell migration from peritoneal cavity to PBMC and LN.

There is abundant evidence that complement activation, cytokine production and other inflammatory responses occur in sepsis [28]. It is generally accepted that the complement activation product complement 5a (C5a) plays an important inflammatory role in rodents following CLP [29]. C5a exerts its effects by binding the high-affinity C5a receptor (C5aR) and C5L2. C5L2, a putative “default” receptor, has been suggested to play an important role in balancing the biological effect of C5a. For example, recent data have shown that both C5aR and C5l2 cooperatively play functional parts in the setting of sepsis [30]. It has been shown that blockade of C5a or its receptor (C5aR) can inhibit the development of CLP. In this study, we used anti-C5a antibody to treat sepsis and found that anti-C5a effectively reduced CLP development (Fig. 1B) by blocking the C5a effect (Fig. 1A).

Reduction of sepsis by C5a blockade is associated with decreased levels of bacteria, preservation of innate immune functions of neutrophils in the blood, reduced thymocyte apoptosis and greatly improved survival rates [31], [32]. We propose that blockade of C5a can affect IL-12^+^DC cell migration. Our data shows that anti-C5a reduced IL-12^+^DC cells in PBMC and LN of septic mice (Fig. 2). There was an increase of IL-12^+^DC cells detected in the peritoneal cavity of anti-C5a-treated septic mice (Fig. 3). These results suggest that C5a induced IL-12^+^DC cell migration from the peritoneal cavity to PBMC and LN. Our data was in accordance with previous studies suggesting that the complement-activated products C5a is a potent chemoattractant.

After CLP induction the IL-12^+^DC cell population in the peritoneal cavity were largely decreased (Fig. 4). On the other hand, anti-C5a-treated septic mice demonstrated high levels of IL-12 production in the peritoneal cavity (Fig. 3). These results suggest that the production of IL-12 in the peritoneal cavity is negatively associated with the severity of sepsis. In the analysis of cell populations by flow cytometry, we found that IL-12 was mainly expressed by DC cells (Fig. 4). Additionally, the DC-depleted mice demonstrated the lack of IL-12-expressing cells (Fig. 5A). These results suggest that DC cells present the main source of IL-12. Dendritic cells are the main APC and central components of host innate immune system. Furthermore, our study shows that DC-depletion exacerbated the septic process (Fig. 5B). There was an increase of percentage survival DC-depleted septic mice when exogenous IL-12 was administered (Fig. 7). All together, the data suggests that IL-12 secreted by dendritic cells plays a protective role in the peritoneal cavity during sepsis. Previous publications have provide evidence that IL-12 plays a major role in defense mechanisms against bacterial infection, and that deficiency of IL-12 decreases resistance to polymicrobial sepsis caused by CLP [33].

Our current study shows that C5a induced IL-12^+^DC cells migration from peritoneal cavity to periphery blood and lymph node. In addition, these IL-12^+^DC cells induced pathogenic IFNγ^+^Th1 and IL-17^+^Th17 cells in peripheral blood and lymph nodes, whereas IL-12, secreted by DC cells in the peritoneal cavity, elucidated its important properties for protecting against sepsis. In conclusion, C5a regulated IL-12^+^DC migration to induce pathogenic Th1 and Th17 cells in sepsis.
